# Unraveling shared risk factors for diabetic foot ulcer: a comprehensive Mendelian randomization analysis

**DOI:** 10.1136/bmjdrc-2023-003523

**Published:** 2023-11-20

**Authors:** Kangli Yin, Tianci Qiao, Yongkang Zhang, Jiarui Liu, Yuzhen Wang, Fei Qi, Junlin Deng, Cheng Zhao, Yongcheng Xu, Yemin Cao

**Affiliations:** 1 Graduate School, Shanghai University of Traditional Chinese Medicine, Shanghai, China; 2 Diagnosis and Treatment Center of Peripheral Vascular Disease, Shanghai TCM-Integrated Hospital, Shanghai, China; 3 Western Medicine Affiliated to Shanghai University of Traditional Chinese Medicine, Shanghai, China; 4 Second Department of Vascular Anomalies Disease, Shanghai TCM-Integrated Hospital, Shanghai, China; 5 Diagnosis and Treatment Center of Peripheral Vascular Disease, Shanghai University of Traditional Chinese Medicine, Shanghai, China

**Keywords:** diabetic foot, neuropathy and vascular disease, type 2 diabetes

## Abstract

**Introduction:**

Diabetic foot ulcer (DFU) stands as a severe diabetic lower extremity complication, characterized by high amputation rates, mortality, and economic burden. We propose using Mendelian randomization studies to explore shared and distinct risk factors for diabetic lower extremity complications.

**Research design and methods:**

We selected uncorrelated genetic variants associated with 85 phenotypes in five categories at the genome-wide significance level as instrumental variables. Genetic associations with DFU, diabetic polyneuropathy (DPN), and diabetic peripheral artery disease (DPAD) were obtained from the FinnGen and UK Biobank studies.

**Results:**

Body mass index (BMI) emerged as the only significant risk factor for DPAD, DPN, and DFU, independent of type 2 diabetes, fasting glucose, fasting insulin, and HbA1c. Educational attainment stood out as the sole significant protective factor against DPAD, DPN, and DFU. Glycemic traits below the type 2 diabetes diagnosis threshold showed associations with DPAD and DPN. While smoking history exhibited suggestive associations with DFU, indicators of poor nutrition, particularly total protein, mean corpuscular hemoglobin, and mean corpuscular volume, may also signal potential DFU occurrence.

**Conclusions:**

Enhanced glycemic control and foot care are essential for the diabetic population with high BMI, limited education, smoking history, and indicators of poor nutrition. By focusing on these specific risk factors, healthcare interventions can be better tailored to prevent and manage DFU effectively.

**WHAT IS ALREADY KNOWN ON THIS TOPIC**

- This study fills a crucial research gap by employing Mendelian randomization (MR) to investigate causal relationships between genetic variants and diabetic foot ulcers (DFU), shedding light on the risk factors for diabetic lower extremity complications. DFUs have a significant global impact on healthcare systems, with high rates of amputation, mortality, and healthcare costs, but comprehensive MR-based research in this area is lacking.

**WHAT THIS STUDY ADDS**

- This MR-based study reveals that body mass index (BMI) independently contributes to the risk of diabetic lower extremity complications, including DFU, diabetic polyneuropathy (DPN), and diabetic peripheral artery disease (DPAD), regardless of type 2 diabetes and glycemic traits. Moreover, it identifies educational attainment as a protective factor against DFU, DPN, and DPAD. The research also suggests that glycemic traits below the type 2 diabetes threshold may influence DFU and DPN occurrence and indicates potential DFU risk associated with smoking history and indicators of poor nutrition. These findings emphasize the need for tailored healthcare interventions to effectively prevent and manage DFU.

**HOW THIS STUDY MIGHT AFFECT RESEARCH, PRACTICE OR POLICY**

- This study’s findings hold significant implications for diabetic lower extremity complications in research, practice, and policy. Healthcare practitioners can prioritize patients with high BMI, limited education, smoking history, and poor nutrition indicators for targeted care. Policymakers may consider integrating specific educational programs and interventions to address these risk factors and reduce the burden on healthcare systems. The study’s use of MR encourages further exploration of causal relationships in diabetes research, paving the way for more effective and tailored approaches to prevent and manage DFU and related complications.

## Introduction

Diabetic foot ulcer (DFU) is the most severe complication of diabetes. Globally, the average prevalence of DFU is 6.3%, with Europe and the Western Pacific region being the most affected areas.^1^ Amputation and mortality are the most severe outcomes of DFU. Investigations have shown that DFU is the leading cause of non-traumatic amputations worldwide.^2^ Moreover, recent global meta-analyses revealed 1, 3, and 5-year survival rates of 86.9%, 66.9%, and 50.9% for DFU, with the lowest rates in Europe and the Western Pacific region.^3^ Patients with DFU face a 30–40 times higher risk of death after amputation than patients with non-ulcerative diabetes.^4^ Even without amputation, the mortality risk for DFU remains 1.89 times higher than for other patients with diabetes.^5^ In the USA, 13% of patients with DFU account for 30% of the total healthcare expenditure for diabetes.^6^

DFU’s occurrence is closely related to diabetic lower extremity complications, primarily diabetic polyneuropathy (DPN) and diabetic peripheral artery disease (DPAD). Other relevant include diabetic dermal lesions, muscle atrophy, etc.^7^ Implementing a diabetes high-risk foot screening prevention model centered on quickly identifying DPN and DPAD has significantly reduced the risk of DFU.^8^ However, despite substantial investment, the incidence of DFU has continued to rise in recent years. Therefore, a more refined DFU-centered prevention model is an urgent need, requiring answers to several critical questions, including shared factors among diabetes lower limb complications, factors beyond DPN and DPAD that influence DFU occurrence, and the characteristic distribution of influencing factors for ischemic and non-ischemic DFUs.

Although current research partially addresses these questions, particular diabetes complications, such as diabetic kidney disease, have significantly increased the risk of DFU.^9^ Nevertheless, due to study limitations in terms of scale, comprehensive measurements have been challenging, given the complex clinical features of the DFU population. As an emerging epidemiological research approach, Mendelian randomization (MR) effectively addresses the issues above. By leveraging genetic variations predating exposure and outcome occurrences, MR minimizes bias caused by selection and achieves results that mimic a randomized controlled trial. By relying on large-scale genome-wide association studies (GWAS) and the robustness of gene variation grouping, MR identifies causal relationships between outcomes and exposure factors by setting reasonable statistical thresholds and selecting instrumental solid variables. Consequently, researchers have used MR to investigate risk factors for various diseases, including diabetes,^10^ cardiovascular diseases,^11^ myocardial infarction,^12^ and cerebrovascular diseases,^13^ strengthening existing evidence and uncovering previously overlooked potential associations.

In this study, we conducted MR analyses for 85 phenotypes across five categories. By observing causal relationships, we aim to identify potential influencing factors for diabetic lower extremity complications. This study is the first MR study related to DFU, and our findings will provide more reliable evidence for its prevention.

## Research design and methods

### Genetic instruments selection

Genetic instruments of exposure phenotype include five categories: common diseases, glycemic traits, anthropometric, behavior, and laboratory examinations. The selection of exposure data was based on summary data from single-ancestry GWAS studies, ensuring less than 50% overlap in population distribution between exposure and outcome data. Specific information about sample size, population, and Pubmed ID for each phenotype can be found in the online supplemental file.

10.1136/bmjdrc-2023-003523.supp1 Supplementary data

This study used single nucleotide polymorphisms (SNPs) as instrumental variables (IVs). SNPs are specific positions in the DNA sequence where a single base pair variation occurs. To ensure robust effect estimation, IVs must satisfy the three core assumptions: (1) the relevance assumption—the genetic variations used as IVs are related to the exposure; (2) the independence assumption—no correlation between IVs and the outcome; (3) the exclusion restriction assumption—the IVs are unrelated to any confounding factors other than the exposure. The p value threshold of IVs is 5e-8.

To address endogenous horizontal pleiotropy resulting from close physical distances on the chromosome, IVs underwent PLINK clump data correction to account for inherent linkage disequilibrium, with a requirement of r^2^<0.001 and a distance greater than 10 000 kb. The study excluded the IVs showing solid correlations with outcomes and confounding factors (p<1e-5) by using the PhenoScanner database and removed IVs with a minor allele frequency of less than 1%. Additionally, to minimize the risk of type II errors, IVs with F-statistic values less than 10 were excluded in advance. The statistical power of the results was estimated using the website(https://shiny.cnsgenomics.com/mRnd/) after the analysis.

### Data source of outcomes

The summary data of outcomes are from two national biobanks, the UK Biobank (UKBB) and FinnGen (FINN), and the related information is shown in table 1.

**Table 1:** Outcome dataset information

Trait report name	Ancestry	Year	Case (n)	Control (n)	Sample (n)	Category
Diabetic neuropathy/ulcer	EUR	2020	130	420 343	420 473	Binary
Diabetic peripheral atherosclerosis	EUR	2021	111 970	225 597	168 832	Binary
Diabetic polyneuropathy	EUR	2022	1048	374 434	375 482	Binary

EUR, European.

The DFU outcome data were derived from the PAN UKBB initiative in 2020 using genetic summary data from 420 473 individuals of European ancestry from the UKBB. Among them, 130 cases had DFU, while 420 343 individuals served as controls. They were diagnosed based on trained nurse investigations and self-reports.

The DPN outcome data came from the FINN R9, including 1048 DPN cases and 374 434 controls. DPN was defined based on electronic medical records and International Classification of Diseases (ICD) codes.

The DPAD outcome data were from the FINN R7, with 111 970 DPAD cases and 225 597 controls. DPAD was defined based on electronic medical records and ICD codes.

### Statistical analysis

This study used R V.4.1.3 software for statistical analysis and the R packages *TwoSampleMR*, *MR-PRESSO*, *MendelianRandomization*, *RMediation*, and *meta*.

All phenotypes performed single-variable MR (SMR) analysis. For phenotypes with only one SNP, we used the Wald ratio to estimate the effect size. This method uses regression coefficients between the outcome and exposure to calculate the causal effect, providing accurate estimates in single-instrument regression.

The main effect was estimated using the inverse-variance weighted (IVW) method for exposure factors with multiple IVs. IVW performs weighted regression using the inverse variance of the IVs, simulating the results of two-sample MR at the individual level without heterogeneity and pleiotropy, making it the main result.

The study also incorporated several sensitivity analyses. The MR-Egger approach, based on the IVW method, adds an intercept through quadratic fitting, ensuring a weaker assumption premise. In cases where multiple IVs exist, the MR-Egger method can provide valid causal estimates even when horizontal pleiotropy is present. The MR-PRESSO method estimates horizontal pleiotropy by successively eliminating SNP and rerunning the IVW test. It calculates the total residual distance from each IV to the postexclusion IVW effect. This method re-estimates the results that have horizontal pleiotropy after significant outliers are eliminated. Similarly, the ‘except one’ analysis, like the MR-PRESSO method, identifies IVs that significantly influence the overall effect’s significance and direction by successively removing SNPs. The heterogeneity test uses the Cochran’s Q method to compare proportional differences between each IV. A larger Q value indicates more apparent heterogeneity.^14^

A meta-analysis was conducted on standardized effect sizes using a fixed effects model for exposure factors from multiple sources of the same phenotype. The DerSimonian-Laird method estimated the variance of the overall effect, and I^2^ estimated the magnitude of heterogeneity in the meta-analysis, classified as low, moderate, high, or significant heterogeneity using cut-off points of 25%, 50%, and 75%. Meta-analysis results were considered the main results of this study to minimize the loss of statistical power.

The Benjamini-Hochberg method controlled the false discovery rate of multiple hypothesis testing. Exposure factors with corrected p values less than 0.05 were considered to have a ‘significant causal association’ with the outcome, and those with uncorrected p values less than 0.05 but corrected p values greater than 0.05 were supposed to have a ‘suggestive causal association’.

## Result

A total of 148 genetic data sources with 85 phenotypes were included in this study. There are 14 phenotypes which only had single GWAS source (13 phenotypes only had European ancestry and one only had East Asian ancestry). The minimal F-statistic value of IVs is 22.44 and the maximum is 3634.22. The relationship of each trait and outcomes is unidirectional through Steiger test.

### SMR analysis results

**Figure 1:**
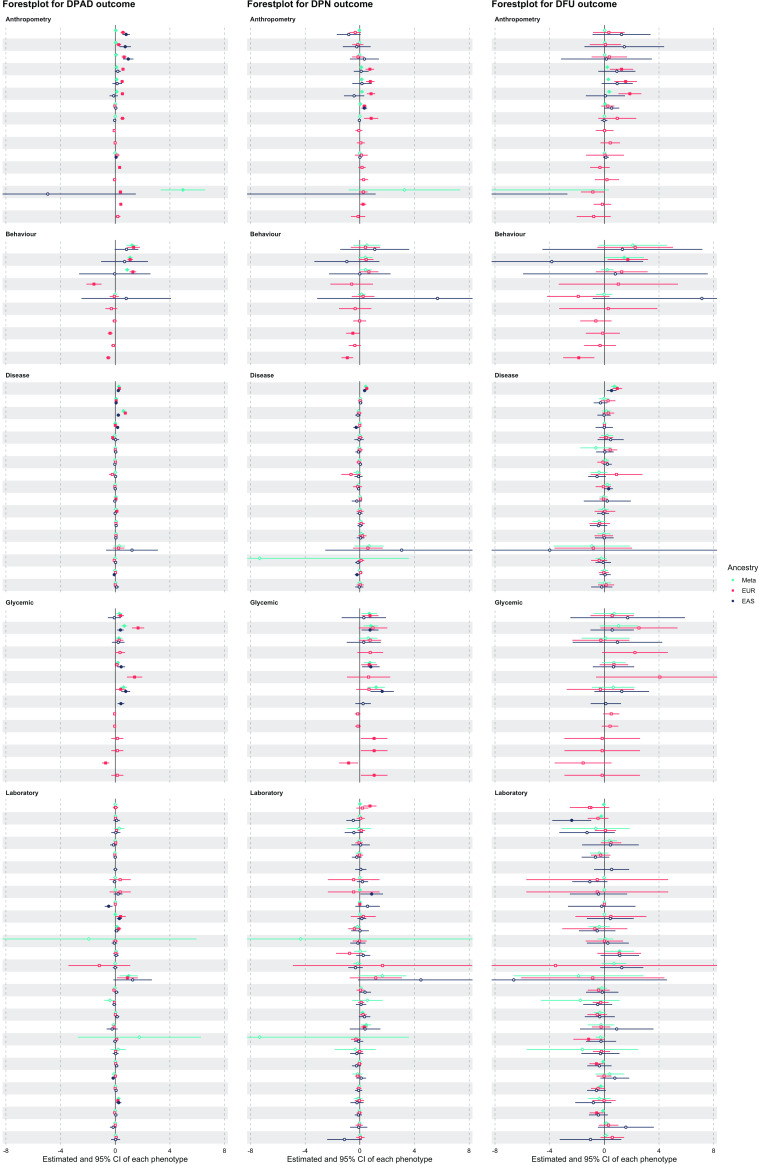
Forest plot for all traits estimated effect on each outcome. DFU, diabetic foot ulcer; DPAD, diabetic peripheral artery disease; DPN, diabetic polyneuropathy; EAS, East Asian; EUR, European.

Figure 1 is a forest plot for beta coefficient of all traits in each outcome.

#### Diabetic foot ulcer

Five phenotypes exhibit significant causal associations with DFU: seven show suggestive causal associations. Body mass index (BMI) is a significant risk factor (OR 1.24, 95% CI 1.09 to 1.42 per kg/m^2^). This correlation remains stable between both sexes, with males (OR 1.43, 95% CI 1.20 to 1.71) having a slightly higher risk than females (OR 1.34, 95% CI 1.16 to 1.54). Creatine kinase (OR 3.05, 95% CI 1.06 to 8.78 per SD unit) and smoking initiation are suggestive risk factors. Compared with individuals who have never smoked, those with a smoking history have a 4.30 times higher risk of DFU (95% CI 1.02 to 18.21).

Years of schooling are the only significant protective factor (OR 0.15, 95% CI 0.05 to 0.49 per SD unit). Total protein (TP) has an adjusted p value close to the statistical threshold (p_adjusted_=0.051) and is a protective factor (OR 0.80, 95% CI 0.69 to 0.93 per g/L). Other suggestive protective factors include mean corpuscular volume (MCV) (OR 0.91, 95% CI 0.84 to 0.98 per fL), mean corpuscular hemoglobin (MCH) (OR 0.78, 95% CI 0.63 to 0.96 per pg), 25-hydroxyvitamin D (25OHD) (OR 0.97, 95% CI 0.94 to 0.99 per nmol/L), and platelet (OR 0.92, 95% CI 0.85 to 0.99 per 10^9^/L).

#### Diabetic polyneuropathy

Nine phenotypes exhibit significant causal associations with DPN, and 15 show suggestive causal associations. BMI is also a significant risk factor (OR 1.12, 95% CI 1.07 to 1.18). The risk is slightly higher in males (OR 1.17, 95% CI 1.10 to 1.26) compared with females (OR 1.15, 95% CI 1.09 to 1.22). Height (OR 1.04, 95% CI 1.03 to 1.06 per cm) and Aspartate aminotransferase (AST) (OR 1.003, 95% CI 1.001 to 1.004 per IU/L) are also significant. All glycemic traits, except 2-hour glucose, show causal associations with DPN. HbA1c (OR 3.32, 95% CI 1.76 to 6.26 per SD unit) and BMI-adjusted fasting insulin (FI) levels (OR 2.24, 95% CI 1.31 to 3.82 per SD unit) are significant. Fasting glucose (FG) is a suggestive risk factor, with BMI-adjusted FG (OR 2.00, 95% CI 1.12 to 3.57) showing a slightly higher risk compared with FI (OR 1.99, 95% CI 1.11 to 3.58). Years of schooling (OR 0.46, 95% CI 0.30 to 0.70) are also a significant protective factor for DPN, while the hardest math class (OR 0.60, 95% CI 0.37 to 0.99 per SD unit) shows suggestive protective effects.

#### Diabetic peripheral artery disease

Thirty phenotypes exhibit significant causal associations with DPAD: three show suggestive causal associations. BMI is also significant for DPAD (OR 1.10, 95% CI 1.08 to 1.12). The risk is slightly higher in males (OR 1.11, 95% CI 1.09 to 1.14) than in females (OR 1.10, 95% CI 1.08 to 1.12). Hypertension (OR 1.003, 95% CI 1.001 to 1.004), coronary artery disease (OR 1.82, 95% CI 1.70 to 1.95), and atrial fibrillation (OR 1.06, 95% CI 1.02 to 1.11) are significant risk factors.

All blood pressure traits are the risk factors for DPAD. Among them, pulse pressure is the most significant (OR 1.06, 95% CI 1.04 to 1.07 per mm Hg), while systolic blood pressure (OR 1.03, 95% CI 1.02 to 1.04) and diastolic blood pressure (OR 1.03, 95% CI 1.01 to 1.04) exhibited similar effects.

Smoking traits are the risk factors for DPAD. Compared with never smokers, smoking initiation was associated with a 2.95 times increased risk (95% CI 2.43 to 3.59), and for ex-smokers, current smokers have a 3.40 times higher risk (95% CI 2.25 to 5.13). Every 1 SD unit increase in daily smoking is associated with a 2.40 times higher risk (95% CI 2.03 to 2.82). Moreover, starting smoking at an older age is associated with a more significant reduction in DPAD risk (OR 0.68, 95% CI 0.59 to 0.78 per SD unit).

All glycemic traits are significant, with FI (OR 4.11, 95% CI 2.34 to 7.22) having the most significant effect, followed by BMI-adjusted FI (OR 1.94, 95% CI 1.57 to 2.40), HbA1c (OR 1.83, 95% CI 1.44 to 2.33), 2-hour glucose (OR 1.51, 95% CI 1.18 to 1.93), and BMI-adjusted FG (OR 1.34, 95% CI 1.08 to 1.67).

Uric acid (OR 1.13, 95% CI 1.04 to 1.23 per mg/dL), gamma-glutamyl transferase (OR 1.004, 95% CI 1.002 to 1.01 per IU/L), mean corpuscular hemoglobin concentration (OR 1.28, 95% CI 1.13 to 1.45 per percent), waist circumference (WC) (OR 1.006, 95% CI 1.003 to 1.008 per cm), female WC (OR 1.37, 95% CI 1.23 to 1.53 per SD unit), waist to hip ratio (WHR) (OR 141.67, 95% CI 27.89 to 719.46 per fold), and female WHR (OR 1.50, 95% CI 1.36 to 1.66 per SD unit) are also significant risk factors for DPAD.

Protective factors for DPAD include years of schooling (OR 0.54, 95% CI 0.47 to 0.62), hardest mathematics class (OR 0.69, 95% CI 0.57 to 0.82), and BMI-adjusted insulin sensitivity index (OR 0.49, 95% CI 0.38 to 0.64 per SD unit).

The sensitivity analysis detected heterogeneity in a few exposures related to the DPN in FINN, specifically from European origin BMI, bipolar affective disorder, serum uric acid, lymphocyte count, and unadjusted FI levels. No significant level of horizontal pleiotropy was detected in others. Some exposure factors showed a degree of heterogeneity across different outcomes, but the Weight median (WM) results indicated their directions were consistent with the IVW results. The MR-PRESSO analysis revealed that outliers existed for certain exposure factors across all outcomes. However, when these outliers were eliminated, the results remained consistent with the primary outcomes, with no significant changes in statistical significance or effect direction. After removing the outliers, the CI of the estimated effect was slightly smaller than the primary results. The leave-one-out analysis did not identify any IV that could significantly change the results. The results of the sensitivity analysis, along with the leave-one-out plot, funnel plots, and scatter plots, can be found in online supplemental files S1–S3.

10.1136/bmjdrc-2023-003523.supp2 Supplementary data

### Multivariable Mendelian randomization results

After type 2 diabetes (T2D) correction, BMI maintained significant associations with DFU, DPN, and DPAD. In the T2D population, for every 1 kg/m^2^ increase, the risk increased by 1.17 times (95% CI 1.002 to 1.36, p=0.047), 1.06 times (95% CI 1.01 to 1.12, p=0.029), and 1.04 times (95% CI 1.02 to 1.06, p<0.01) of DFU, DPN, and DPAD.

After BMI correction, the protective effects of years of schooling on DFU (p=0.21) and DPN (p=0.17) were no longer statistically significant but still maintained a meaningful protective causal relationship with DPAD (OR 0.69, 95% CI 0.58 to 0.84, p<0.01). For DFU, after considering BMI levels, the protective effect of 25OHD on DFU was not significant (p=0.97). Similarly, the association between atrial fibrillation (AF) and DPAD was insignificant after BMI correction (p=0.86).

We used FG, FI, and HbA1c as the covariates in the multivariable Mendelian randomization (MVMR) model. After adjusting for FG, BMI still maintained a significant causal association with DFU (OR 1.33, 95% CI 1.16 to 1.54), DPN (OR 1.16, 95% CI 1.11 to 1.22), and DPAD (OR 1.09, 95% CI 1.07 to 1.11) with a slight reduction in the effect size. After adjusting for FI, BMI still maintained a significant causal relationship with DFU (OR 1.35, 95% CI 1.17 to 1.56), DPN (OR 1.16, 95% CI 1.10 to 1.22), and DPAD (OR 1.09, 95% CI 1.07 to 1.12). Similarly, after adjusting for HbA1c, BMI still maintained a significant causal association with DFU (OR 1.36, 95% CI 1.19 to 1.56), DPN (OR 1.15, 95% CI 1.10 to 1.21), and DPAD (OR 1.09, 95% CI 1.07 to 1.11).

The results of the sensitivity analysis indicated that after incorporating BMI, T2D, and blood glucose characteristics separately into the multivariate model, no significant horizontal pleiotropy was detected in the MR-PRESSO results.

## Discussion

### Body mass index

Our results demonstrate that BMI is simultaneously the only significant risk factor for DFU, DPN, and DPAD. This association remains stable after controlling for T2D, glycemic traits, and educational attainment (EA). As an essential risk factor for various diseases, a meta-analysis of MR studies showed that BMI has causal associations with T2D, aortic valve stenosis, heart failure, stroke, peripheral artery disease, death due to chronic obstructive pulmonary disease (COPD), and kinds of tumors.^15^ Observational studies also confirm that BMI increases the risk of T2D and raises the risk of microvascular and cardiovascular complications in diabetes.^16–18^

As the most severe diabetic complication, recent meta-analyses have shown that BMI>24.5 is an independent risk factor for new-onset DFU.^19^ For the first time, this study confirms the causal relationship between BMI and DFU through genetic prediction, demonstrating that BMI is an essential factor contributing to both ischemic and non-ischemic DFU development. The research also found that the effect of BMI increases on the risk of DFU, DPN, and DPAD is more pronounced in males compared with females. This finding is consistent with previous observational studies, where males were identified as an independent risk factor for new-onset DFU and associated with amputation risk.^20 21^ The MVMR results further confirm that BMI remains independently associated with increased risks of DFU, DPN, and DPAD in the T2D population, further strengthening the evidence of the association between BMI and DFU.

The relationship between BMI and diabetes is tightly linked through insulin resistance, oxidative stress, mitochondrial homeostasis imbalance, and other mechanisms,^22^ and this effect usually exceeds the time limit prescribed by clinical observation. Because the effect estimated by MR has a significant time accumulation effect, the effect estimate provided is closer to the actual situation between BMI and diabetes.

### Educational attainment

EA is the only protective factor with a robust causal association with all three outcomes. DFU exhibits significant economic inequality in the overall population, with a higher incidence occurring in people with limited education, lower incomes, and remote areas.^23^ This study confirms a causal relationship between EA and diabetic lower extremity complications. The correlation between EA and diabetes knowledge level and the close link between EA and BMI can explain it. The research shows that compared with individuals with higher education, those with limited education levels have more lacking self-awareness of their BMI.^24^ A generational study in Finland found that EA negatively correlates with BMI at the individual level and maintains a 60% correlation across generations. Moreover, within families, the correlation between education level and BMI is more potent in offspring compared with the influence of their parents’ BMI.^25^

However, the causal association becomes non-significant after controlling for BMI and T2D, suggesting that the protective effect of education on DFU and DPN is more likely to be mediated through its impact on BMI levels. However, for DPAD, the level of education still exerts its influence through pathways independent of BMI. Analysis based on the ADVANCE trial (Action in Diabetes and Vascular Disease-PreterAx and DiamicroN Controlled Evaluation) found that the duration of diabetes is independently associated with diabetic microvascular complications, while the age of diagnosis is only related to macrovascular complications.^26^ Therefore, DPAD interacts with cardiovascular risk factors in addition to diabetes-related factors.

### Smoking

Smoking traits are associated with an increased risk of DFU and DPAD. Tobacco use has been confirmed as a risk factor for peripheral artery disease (PAD) and exhibits a significant cumulative effect.^27^ The results of the SMR analysis confirm a dose-dependent effect of tobacco on DPAD. This study also found an association between DFU risk and smoking history, although the significance is lost after multiple comparisons. However, observational studies have shown that smoking is a risk factor for DFU incidence and severity and an independent risk factor for major amputation.^28 29^ Tobacco’s impact on DFU is multidimensional, as DPAD-induced limb ischemia increases the difficulty of treating and significantly raises the amputation risk. The coexistence of PAD also substantially increases the risk of cardiovascular death in patients with DFU.^30^ Smoking also promotes obesity and insulin resistance, further exacerbating the severity of diabetes.

Although no smoking traits have a causal relationship with DPN in this study, a meta-analysis conducted in 2015 showed a 42% increased risk of DPN associated with smoking.^31^ However, this evidence was reported as low intensity due to the limitations of the included studies. Taken together with our results, the association between smoking and DPN may not be direct.

### Glycemic traits

In this study, glycemic traits, insulin sensitivity index, insulin fold count, and proinsulin levels did not directly correlate with DFU. The genetic data for these glycemic traits were obtained from a cohort consisting of non-diabetic individuals, potentially elucidating the absence of a demonstrated causal relationship between glycemic traits and outcomes. At the same time, DFU typically occurs in patients with diabetes with poorer glucose control. In many studies, the average HbA1c level in DFU groups is often higher than 9%.^32^ The lower average values of glycemic traits could be a primary reason for the lack of significance. Observational studies demonstrated that higher FG and HbA1c levels were independent risk factors for delayed wound healing and amputation risk in DFU.^33^

HbA1c is considered the golden standard for glucose control. A global longitudinal cohort has shown that higher HbA1c levels are the main risk factors for diabetic macrovascular and microvascular complications, and the rate of satisfied HbA1c control in T2D groups is less than 40%. Some studies suggest that maintaining HbA1c levels between 7.0% and 7.7% can significantly reduce the risk of complications.^34^ In the results of this study, HbA1c levels increased the chances of DPN and DPAD significantly for each SD unit (approximately 0.3%). The risk increase in DPN was nearly three times that in DPAD. Observational studies have also found that diabetic microvascular complications are more common than macrovascular complications and more influenced by age, glucose control, and diabetes duration.^35^

FG and FI also have a similar impact to DPN and DPAD. A clinical control study observed that about 19.4% of patients with impaired glucose tolerance reported peripheral neuropathic symptoms.^36^ While an earlier small-scale survey did not find a significant difference in DPN incidence between glucose-impaired individuals and the general population,^37^ recent researchers generally agree that DPN occurrence is a continuous process along with impaired glucose and diabetes diagnosis.^38^ Therefore, early intensified glucose intervention is crucial for preventing diabetic microvascular complications. This study supports this view, suggesting that avoiding diabetic complications may require stricter blood glucose control standards than current diabetes diagnosis.

The study did not find a causal relationship between postprandial blood glucose and DPN. Although some small clinical observations found that painful DPN was associated with higher postprandial blood glucose levels in patients with diabetes with normal FG levels but high HbA1c levels, this association was not observed in painless DPN.^39^ Since this trial did not directly monitor postprandial glucose levels, further research must confirm the causal relationship between postprandial blood glucose traits and DPN.

### Nutrition

Nutritional factors have unique causal relationships with DFU worth noting. This study found that TP, MCV, and MCH are only associated with a decreased risk of DFU. A single-arm study that evaluated the nutritional status of DFU using serum TP and hemoglobin levels found that malnutrition is associated with an increased risk of worse outcomes in patients with DFU.^40^ Another study also confirmed that patients with DFU with amputation and death have lower TP and hemoglobin levels.^41^ These researchers believe that low nutritional status is a consequence of chronic wounds and infections. Observational studies have found that supplementing trace elements in patients with type 2 diabetes mellitus leads to a significant increase in TP levels and a significant reduction in the incidence of infections.^42^ TP can assess overall nutritional status and correlates with antioxidant capacity, immune defense, and dermal repair capabilities. In 2021, a small-scale survey found significantly lower TP levels in the diabetes complication group.^43^

Red blood cell characteristics are related to microvascular changes. In a study for diabetic retinopathy (DR) with non-anemia diagnosis, researchers observed that MCV and red cell width are associated with the progression of DR.^44^ Another cross-sectional iron-deficiency anemia survey and retinal vasculopathy found that MCH and MCV are related to retinal capillary density.^45^ Red blood cell deformability can affect blood viscosity, leading to oxidative stress, a mechanism that has been confirmed to be associated with the progression of cardiovascular disease.^46^

Combined with the findings of this study, the absence of protective factors, including TP, MCV, and MCH, may already exist before the occurrence of DFU and influence its development. Considering the success of foot care education as a solid preventive measure in reducing the incidence of DFU, it seems necessary to pay attention to strengthening interventions for patients with diabetes with low nutritional levels.

This study’s strengths encompass its pioneering utilization of genetic prediction to explore major diabetic lower extremity complications, allowing a comparison of shared and distinct risk factors. This informs targeted prevention strategies for DFU. Nonetheless, limitations emerge from outcome data predominantly sourced from European populations, constrained by study sizes, constraining result generalizability. Modest outcome sample sizes might under-represent positive factors, particularly as continuous variables like blood glucose traits are primarily derived from healthy individuals. Although the MVMR method adjusts for BMI and T2D, it introduces potential collinearity risk. Furthermore, the inherent time lag in MR could exaggerate effects for specific non-stationary variables. Mitigating these constraints, the study has implemented a more rigorous methodology, bolstering the credibility of reported findings.

## Conclusion

The findings of this study at the genetic prediction level suggest that an increase in BMI is significantly associated with an elevated risk of major lower limb complications in diabetes, and this association is independent of blood glucose characteristics. Higher educational levels are significantly associated with a reduced risk of major lower limb complications in diabetes, and this protective effect is likely mediated through BMI. Increases in FG, FI, and HbA1c below the threshold for T2D diagnosis also show a causal relationship with the occurrence of DPN and DPAD. Low nutritional status may be an essential risk factor for DFU in patients with DPN and DPAD.

In conclusion, for patients with diabetes with high BMI, limited education, smoking history, and poor nutritional status, stricter blood glucose control and enhanced diabetes foot prevention are necessary.

## Data Availability

All data relevant to the study are included in the article or uploaded as supplementary information.
